# An evaluation of time series summary statistics as features for clinical prediction tasks

**DOI:** 10.1186/s12911-020-1063-x

**Published:** 2020-03-05

**Authors:** Chonghui Guo, Menglin Lu, Jingfeng Chen

**Affiliations:** 1 Institute of Systems Engineering, Dalian University of Technology, No. 2 Linggong Road, Ganjingzi District, Dalian, 116024 People’s Republic of China; 2 Health Management Center, The First Affiliated Hospital of Zhengzhou University, No. 1 Longhu central ring road, Zhengzhou, 450052 People’s Republic of China

**Keywords:** Patient representation, Clinical prediction tasks, Genetic algorithm, Summary statistics

## Abstract

**Background:**

Clinical prediction tasks such as patient mortality, length of hospital stay, and disease diagnosis are highly important in critical care research. The existing studies for clinical prediction mainly used simple summary statistics to summarize information from physiological time series. However, this lack of statistics leads to a lack of information. In addition, using only maximum and minimum statistics to indicate patient features fails to provide an adequate explanation. Few studies have evaluated which summary statistics best represent physiological time series.

**Methods:**

In this paper, we summarize 14 statistics describing the characteristics of physiological time series, including the central tendency, dispersion tendency, and distribution shape. Then, we evaluate the use of summary statistics of physiological time series as features for three clinical prediction tasks. To find the combinations of statistics that yield the best performances under different tasks, we use a cross-validation-based genetic algorithm to approximate the optimal statistical combination.

**Results:**

By experiments using the EHRs of 6,927 patients, we obtained prediction results based on both single statistics and commonly used combinations of statistics under three clinical prediction tasks. Based on the results of an embedded cross-validation genetic algorithm, we obtained 25 optimal sets of statistical combinations and then tested their prediction results. By comparing the performances of prediction with single statistics and commonly used combinations of statistics with quantitative analyses of the optimal statistical combinations, we found that some statistics play central roles in patient representation and different prediction tasks have certain commonalities.

**Conclusion:**

Through an in-depth analysis of the results, we found many practical reference points that can provide guidance for subsequent related research. Statistics that indicate dispersion tendency, such as min, max, and range, are more suitable for length of stay prediction tasks, and they also provide information for short-term mortality prediction. Mean and quantiles that reflect the central tendency of physiological time series are more suitable for mortality and disease prediction. Skewness and kurtosis perform poorly when used separately for prediction but can be used as supplementary statistics to improve the overall prediction effect.

## Background

Clinical prediction tasks such as patient mortality and disease prediction are highly important for early disease prevention and timely intervention [1, 2]. Patient mortality prediction in intensive care units (ICUs) is a key application for large-scale health data and plays an important role in selecting interventions, planning care, and allocating resources. Accurate assessment of mortality risk and early identification of high-risk populations with poor prognoses followed by timely intervention are key in improving patient outcomes. A preliminary disease diagnosis assists doctors in making decisions. With the goal of accurately predicting clinical outcomes, studies have proposed methods that include scoring systems and machine learning models [3, 4]. The scoring systems for mortality prediction in widely clinical use include the Sepsis-related Organ Failure Assessment (SOFA) [3], the New Simplified Acute Physiology Score (SAPSII) [5], and the Multiple Organ Dysfunction Syndrome (MODS) [6]. However, most scoring systems based on simple logistic regression for patient mortality prediction have limited prediction performance. With the development of machine learning and deep learning models, studies have applied trained models to clinical prediction tasks and achieve better performance compared to earlier approaches [4, 7].

Feature extraction and patient representation are the underlying premise for constructing prediction models; consequently, these factors are important and affect the prediction performance. An increasing number of monitoring devices and laboratory tests in modern ICUs collect multivariate time series data of varying lengths from patients. Variable-length multivariate time series means that more than one physical measurement will be collected from a patient after admission to the ICU and that the sampling frequency of each predictor differs within a given time window. Overall, patient data consisting of physiological measurements have typical characteristics, such as high resolution, varying lengths, noisy values, and system bias, making the extraction of the temporal features of time series challenging. Most of the existing models select specific summary values for each predictor over a given time period and concatenate them to form patient vectors. Statistics are a form of summary values, and studies have shown that summary statistics can reflect the characteristics of time series. Moreover, they have advantages such as simple extraction, high robustness and strong representativeness [8–10]. The features of time series can be divided into three aspects: central tendency, dispersion tendency and distribution shape. The distribution and trends of time series can be reflected by combining multiple summary statistics, thus approximating the original data distribution and reducing the impact of noise on the prediction results.

Existing studies based on machine learning models have mainly used simple summary statistics to summarize time series information, such as maximum and minimum observations, as of physiological time series features. However, this lack of more comprehensive summary statistics leads to a lack of information in physiological time series. In addition, using only the maximum and minimum statistics to indicate patient features fails to provide adequate explanations. Despite the likelihood that more comprehensive features would have clinical implications, few existing studies have experimentally evaluated which summary statistics can best represent physiological time series. In this paper, we report an exhaustive set of results based on different combinations of summary statistics used as features of physiological time series for three clinical prediction tasks. The contributions of this study are twofold: on the one hand, we summarize and use 14 statistics as options for physiological time series representation compared with previous studies that used only a few statistics. On the other hand, we experimentally evaluate the performance of different summary statistics as features of physiological time series for different prediction tasks and obtain many conclusions that have practical implications and can provide guidance for subsequent related research.

The remainder of this paper is arranged as follows. First, we outline the related works. Second, we describe our method and its details and then present the experiments and results. Next, we discuss the results of the previous section. Finally, conclusions and future prospects are provided in the last section.

## Related works

### Methods for representing physiological time series

The most common method for representing physiological time series is to summarize the changing features of data contained in predictors using summary features and concatenate them as representative of a patient. Such statistics are simple and easy to calculate and have wide applications. Some studies also adopt the first measurement of predictors as the characteristic value of time series. The statistics used in some of the existing studies are listed in Table 1. From Table 1; these include maximum, minimum and mean values, which are widely used. One reason for their wide use is that these statistics are easy to acquire. Another is that experts tend to believe that the maximum and minimum observations reflect the normality or abnormality of the patient index, while the mean value reflects the average fluctuation range of the index over a period of time. A few studies have attempted to characterize time series features using statistics such as standard deviation, median and skewness.

**Table 1:** Statistics used in existing research works

No.	Research works	Min	Max	Mean	First	Others
1	Pollack M M, Patel K M, et al. (1996) [11]	✓	✓			
2	Ribas V J, Lpez, et al. (2011) [12]			✓		
3	Fialho A S, Cismondi F, et al. (2012) [13]			✓		
4	Bosnjak A, Montilla G (2012) [14]	✓	✓	✓		std
5	Wiens J, Horvitz E, et al. (2012) [15]	✓	✓	✓		std
6	Eren Gultepe, Jeffrey P Green, et al. (2013) [16]			✓		std, CV, median, IQR
7	Pirracchio R, Petersen M L, et al. (2015) [17]	✓	✓			
8	Lee J, Maslove D M, et al. (2015) [18]	✓	✓			
9	NM Arzeno, KA Lawson, et al. (2015) [19]	✓	✓			
10	Lipton Z C, Kale D C, et al. (2015) [20]			✓		
11	Lee J, Dubin J A, et al.(2016) [21]				✓	
12	Awad A, Baderelden M, et al. (2017) [22]	✓	✓			
13	Morid M A, Sheng O R L, et al.(2017) [23]	✓	✓	✓	✓	median
14	Harutyunyan H, Khachatrian H, et al. (2017) [9]	✓	✓	✓	✓	std, skew
15	Sherman E, Gurm H, et al. (2017) [24]	✓	✓	✓		
16	Purushotham S, Meng C, et al. (2018) [10]	✓	✓	✓		
17	Mayhew M B, Petersen B K, et al. (2018) [25]	✓	✓	✓		std

In addition to the above studies, many studies have attempted to fully understand the temporal trends hidden in multivariate time series data. Hug et al. considered a comprehensive set of physiologic measurements and manually defined a set of trend patterns [26]. McMillan et al. used temporal pattern mining to discover time series feature patterns [27]. Cohen et al. identified clinically relevant patient physiological states from physiologic measurements based on hierarchical clustering [28]. Yuan et al. applied nonnegative matrix factorization to group trends in a way that approximates patient pathophysiologic states [29]. Compared with these methods, patient representation based on summary statistics is a simple concept that is easy to calculate and can improve the interpretability of the results. However, the above studies based on summary statistics do not provide a clear reason why only these statistics were selected. It can be surmised that these choice were subjective and lack theoretical and experimental support. In addition, relevant research to determine which summary statistics can achieve the best performances for physiological time series is lacking. Therefore, the goals of this paper are to discover statistics that yield important summary performances and thus provide support for these studies and to improve model prediction performance based on representations of these summary statistics.

### Feature selection methods

Datasets containing massive amounts of features can reduce classification accuracy, raise the computational cost and increase the risk of overfitting [30, 31]. Varying length multivariate time series can be characterized by multiple summary statistics; however, some statistics may contain useless or redundant information, and some features may be coupled. If representative features are not selected, algorithm resources will be consumed, but accurate classification results will not be obtained. Thus, it is beneficial to use feature selection mechanisms not only to identify the most representative features but also to reduce the number of features. To select a suitable combination of important summary statistics, feature selection is critical [32]. Previous works used three feature selection categories: filter methods, wrapper methods and embedded methods. Genetic algorithms are classically used for feature selection and have wide applicability because they can overcome the shortcomings of exhaustive methods that have high time complexity. Additionally, the genetic algorithm is a feature selection method of combinatorial optimization that can fully consider the relationships between features and find the most suitable feature combinations. Many previous works have selected features based on genetic algorithms and achieved satisfactory results. Leardi R et al. first proposed that the genetic algorithm can be a valuable tool for solving feature selection problems [33]. Mahdi Mohammadi et al. used a genetic algorithm to identify the most significant features of EEG signals and find their diagnostic value for depression [34]. Dino et al. combined a genetic algorithm with gene expression data to classify gene expression data in two steps [35]. Lei et al. proposed a new electrocardiograph pattern recognition method by combining a genetic algorithm with a support vector machine [36].

## Method

Clinical prediction tasks include mortality, length of hospital stay, and disease prediction. The distribution characteristics of physiological time series are the manifestations of physiological states, including dispersion tendency, central tendency, and distribution shape, and these correspond to multiple statistics. By comparing the effects of different statistical combinations on different prediction tasks, the commonalities and differences of the optimal statistical combinations can be found, which can guide subsequent prediction tasks. The premise for finding the best combination of statistics is global search; however, global search is laborious and difficult in practice. This paper considers a feature selection method based on combinatorial optimization, that is, using the genetic algorithm to find the best combinations of statistics.

### Identification of the distribution features of physiological time series

To characterize the time series distribution features of different predictors, it is critical to explore many different aspects of the data distribution. Based on statistical theory and existing research, this paper approximates the original data distribution by analysing the central tendency, the dispersion tendency and the distribution shape of each predictor. The central tendency reflects the representative value of the general level of the data or the central value, including statistics such as the mean, median, mode and quantile. The dispersion tendency of the distribution reflects trends describing how far the data are from the central value, including statistics such as maximum, minimum, standard deviation, coefficient of variation, range and interquartile range. The shape of the distribution reflects whether the distribution is symmetrical, the degree of skewness and the flatness of the distribution, including statistics such as skewness and kurtosis.

**Fig. 1:**
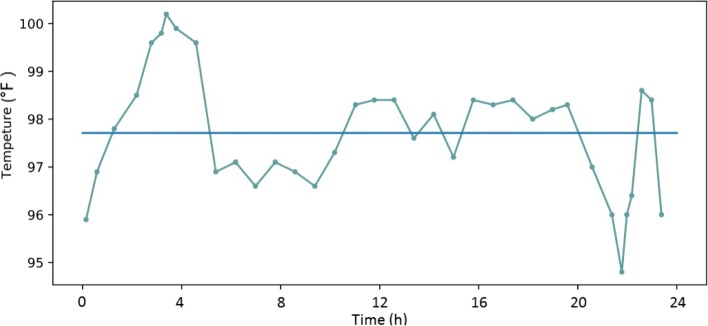
Temperature fluctuation of a patient within 24 hours of admission to the ICU. The straight line represents the mean temperature within 24 hours

Figure 1 shows the temperature fluctuation of a patient within 24 hours of admission to the ICU. The minimum and maximum values reflect the range of temperature change of the patients and can reflect the trend of the data from the centre value. The mean value reflects the average temperature of the patients over 24 hours and can reflect the degree to which the data distribution aggregates to its centre value. Furthermore, the mode reflects the temperature value that appears most frequently within the 24 hours. The median reflects the average value, and the quantile reflects values in a specific position. The range and interquartile range reflect the degree of difference among the whole data distribution. The variance and standard deviation reflect the dispersion degree of the temperature distribution and the stability of the temperature data: a larger variance indicates that the patient’s temperature fluctuates widely, which may indicate that the disease is more severe. The coefficient of variation also reflects the degree of discreteness of the data. However, the central tendency and the dispersion tendency of the temperature distribution cannot reflect the order of temperature measurements; therefore, the shape of the distribution should be considered. The shape of the distribution can reflect the evolution of the disease. Skewness can reflect the symmetry of the data distribution. Generally, the symmetry of the data distribution can be understood as the stability of the temperature change. Both left and right skewness can reflect changes in temperature. Kurtosis reflects sharpness of the peak and the peak degree of the data distribution and reveals the fluctuation trend and the patients’ physiologic state.

The summary statistics used in this study included the 13 statistics mentioned above, namely, minimum (min), maximum (min), mean, standard variation (std), median, lower quartile (Q1), upper quartile (Q3), mode, range, interquartile range (IQR), coefficient of variation (CV), skewness (skew) and kurtosis (kurt). Based on previous works, the first measurement (first) is also added.

### Selection of best statistical combination based on the genetic algorithm

To explore the impact of different combinations of statistics on prediction performance and find the optimal combination, we formalize the problem. Let *V*={*V*_1_,*V*_2_,⋯,*V*_*P*_} represent a collection of *P* multivariate time series. Series *V*_*i*_ consists of a multidimensional time series of *m* variables, and the time series of each variable *j* has *n*_*j*_ observations. For a variable-length time series, *n*_*j*_ may differ for each variable *j*. *V*_*i*_ can be written as follows:

1 $$ V_{i}=\{V_{ijt}\}, j=1,2,\cdots,m;\,t=1,2,\cdots,n_{j}.  $$

The *j*-*th* component of the *i*-*th* time series, that is, $\phantom {\dot {i}\!}V_{i,j}=\{V_{ij1},V_{ij2},\cdots,V_{ijn_{j}}\}$, is a univariate time series. For every univariate time series *V*_*ij*_, the different variables have different dimensions (observations), but every time series can be represented and transformed into *L* summary statistics extracted from the time series. In this paper, according to the 14 statistics mentioned, we set *L*=14.

Multiple clinical predictors with different sampling frequencies from multiple patients are collected in the ICU. Thus, *V* is a set of time series of varying length multivariate time series. Specifically, in Formula (1), *P* represents the number of patients, *m* corresponds to predictor dimensions such as heart rate, blood pressure, temperature and other vital signs and laboratory predictors and *t* is the time measurement point, and the length of *t* differs for different predictor sampling frequencies. Thus, *V*_*ijt*_ denotes the *t*-*th* measurements of the *j*-*th* predictor in the *i*-*th* patient. Because of the different sampling frequencies of different predictors in different patients, the total lengths of the vectors obtained by concatenating them differ. We can summarize the measurements of different variables by statistics of fixed numbers and concatenate them to obtain vectors of the same length for patients. The time series of patient *i* after extracting the time series features using the *L* summary statistics can be expressed as follows:

2 $$ V_{i}'=\{V_{ijk}\}, j=1,2,\cdots,m;\, k=1,2,\cdots,L.  $$

Note that different statistics have specific statistical meanings. Some problems, such as information overlap, may exist among the statistics. Not all the statistics may perform well for prediction; thus, using all the statistics directly to represent a patient will increase the modelling complexity and can lead to overfitting. Let binary variable *x*_*k*_ denote whether statistic *k* is selected in the best combination, that is,

3 $$ x_{k}= \left\{\begin{array}{ll} 0& \text{, statistic {k} is not selected;}\\ 1& \text{, statistic {k} is selected.} \end{array}\right.  $$

Then, the selection vector *X* of the best combination of statistics can be expressed as

4 $$ X=\left(x_{1},x_{2},\cdots,x_{L}\right),  $$

and thus, the representation of patient *i* after statistical selection can finally be expressed as

5 $$ V_{i}'=\{V_{ijk}\vert x_{k}\ne 0\},\,j=1,2,\cdots,m;\,k=1,2,\cdots,L.  $$

To select the combination of statistics that best reflects the physiological time series, we regard the selection vector *X* as an unknown parameter and construct an objective function to solve the optimization problem. The optimal objective function can be written as follows:

6 $$ {\begin{aligned} \underset{X}{\max} E\left(y_{i},f\left(V_{ijk}|x_{k}\ne 0\right)\right),\,i=1,2,\cdots,P;\\ j=1,2,\cdots,m;\,k=1,2,\cdots,L, \end{aligned}}  $$

where *E* is an evaluation function used to measure the prediction performance; in this study, the area under the receiver operating characteristic curve (AUROC) is chosen in this paper. Here, *y*_*i*_ is the true label of the patient in different prediction tasks, and *f* is the prediction model, which is the random forest algorithm in this study. Because the objective function in Formula (6) cannot be written using explicit expression levels, the simplest and most direct way to find the optimal solution of *X* is to adopt a global search strategy, that is, to find the prediction effect of all statistical combinations and then select the optimal combination. However, the time complexity of this method is *O*(2^*n*^−1), which has practical limitations. The purpose of this paper is to evaluate which statistical combination is most effective for time series representation, and the final result of feature selection is a combination of statistics (such as [minimum, maximum and mean]). The optimal combination can be achieved by chromosome coding in a genetic algorithm. The genetic algorithm is a combinatorial optimization algorithm that approximates a global search; it can fully consider the relationships between features and find the most suitable feature combination.

The parameter settings in the genetic algorithm are as follows. (1) Coding and decoding: Because the selection vector of summary statistics is a binary variable, we use binary coding, and no decoding process is needed. (2) Population: We select the size of the population as 20, and the initial population is generated randomly. (3) Fitness function: In this paper, we select the AUROC as the fitness function to select the feature subset with a better classification effect. The fitness function corresponds to *E* in Formula (6). (4) Genetic operators: We use the roulette wheel selection scheme as the selection strategy, single point crossover with a probability of 0.6 as the cross strategy and uniform mutation with a probability of 0.1 as the mutation strategy. (5) Termination condition: To determine the convergence of the algorithm adaptively during the iteration process, the termination condition for the genetic algorithm used in this paper combines the maximum genetic algebra with the stationary fitness value. When the continuous fluctuation range of the fitness value is less than the specified threshold or the genetic algebra is larger than the specified algebra, the solution of the algorithm is complete.

To avoid optimistically biased performance estimates from conducting feature selection on the full dataset, we refer to previous work by Ozcift and Gulten, who embedded a genetic algorithm for feature selection into Bayesian network classifier training using a nested cross-validation approach [37]. The general flow of feature selection with the genetic algorithm is given in Table 2. The feature selection based on the genetic algorithm is embedded in a 5-fold cross-validation. For each fold of test data, a set of summary statistics will be obtained by the genetic algorithm; thus, five groups of summary statistics will be obtained under 5-fold cross-validation. Then, based on the summary statistics of each group, the random forest model is used for prediction, and the mean and standard error of the metrics index is taken as the experimental result.

**Table 2:** The general flow of feature selection by the genetic algorithm

Divide data into k=5 folds
for k=1 to 5
Assign
A = test data (1 fold reserved for random forest)
B = train data (3 folds train for random forest)
C = validation data (1 fold validation for random forest)
Repeat for train and validation data
step 1: Encode features as binary chromosomes
step 2: Generate a population of 20 chromosomes randomly
step 3: Evaluate AUROC of random forest algorithm for step 2
step 4: Determine if termination conditions are met
if yes:
Terminate
else:
step 5.1: Apply Single point crossover with probability
of 0.6
step 5.2: Apply uniform mutation with probability of 0.1
step 5.3: Calculate AUROC of new chromosomes by
random forest and compare it with step 3
step 5.4: Select best chromosomes with highest fitness
step 5.5: Replace chromosomes with lowest fitness,
back to step 4
Train random forest with data (B+C) based on statistics obtained by
the genetic algorithm
Test random forest with data (A)
Calculate AUROC for fold k
End for
Calculate average AUROC for 5 folds

## Experiments and results

We explored the performances of different statistical combinations for different clinical prediction tasks, including patient mortality, length of hospital stay and disease prediction, and obtained the optimal statistical combination based on a genetic algorithm. Then, we analysed the results to find the commonalities and differences of the optimal combinations under different tasks.

### Dataset and preprocessing

We used the MIMIC-III dataset collected from a variety of ICUs between 2001 and 2012 [38]. MIMIC-III is a large, freely available critical care database developed by the Laboratory for Computational Physiology of Massachusetts Institute of Technology (MIT). The database integrates deidentified, comprehensive, health-related data of 58,976 admissions admitted to the ICU of the Beth Israel Deaconess Medical Center (BIDMC) in Boston, Massachusetts.

To reflect the universality of the results, we did not target patients with a certain disease, but accepted all patients. After removing duplicates, we obtained a total of 42,145 admission records; patients less than 15 years of age were excluded. To prevent possible information leakage and to ensure similar experimental settings compared with related works, we used only the first ICU admission for each patient [39]. In the MIMIC-III database, bedside monitoring data, laboratory test data, input events and output events all consist of time series with time tags. The data for the predictors selected in this paper came from three tables: chartevents, labevents and outputevents. Following the related research, we chose the predictors used in SAPS II, as shown in Table 3 [10, 17, 21]. For each predictor, we used raw data instead of calculated data. For example, we treated GCSVerbal, GCSMotor, and GCSEyes from the Glasgow Coma Scale (GCS) score as separate features. All the extracted predictors shown in the table came from the first 24 hours after the patient was admitted to the ICU.

**Table 3:** Predictors used in the experiments

Feature	Item ID	Item Name	Table
Glasgow Coma Scale	184	GCSEyes	chartevents
	220739	Eye Opening	chartevents
	454	GCSMotor	chartevents
	223901	Motor Response	chartevents
	723	GCSVerbal	chartevents
	223900	Verbal Response	chartevents
White Blood Cells Count	51301	White Blood Cells	labevents
	51300	WBC Count	labevents
Potassium Level	50971	Potassium	labevents
	50822	Potassium, whole Blood	labevents
Po2	50821	pO2	labevents
Serum Bicarbonate Level	50882	Bicarbonate	labevents
Sodium Level	50983	Sodium	labevents
Urea Nitrogen (Bun)	51006	Urea Nitrogen	labevents
Bilirubin, Total	50885	Bilirubin, Total	labevents
Temperature	678	Temperature_F	chartevents
	223761	Temperature_Fahrenheit	chartevents
	676	Temperature_C	chartevents
	223762	Temperature_Celsius	chartevents
Urine Output	40055	Urine Out Foley	outputevents
FiO2	223835	Inspired O2 Fraction	chartevents
	190	FiO2 Set	chartevents
Heart Rate(HR)	211	Heart Rate	chartevents
	220045	Heart Rate	chartevents
Systolic Blood Pressure(SBP)	220179	Noninvasive Systolic Blood Pressure	chartevents
	455	NBP[Systolic]	chartevents
Age	-	-	patients
Admission_Type	-	-	admissions

**Fig. 2:**
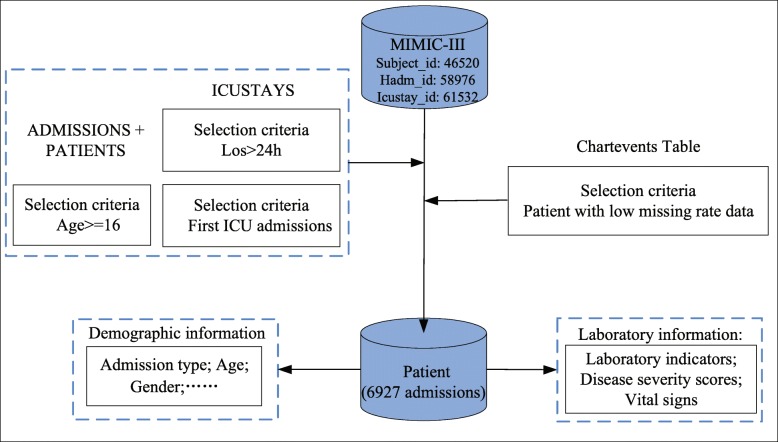
Patient cohort selection inclusion criteria and data extraction process. Adult patients at their first hospital admission with a low missing data rate were selected as the patient cohort, and then, the clinical data of these patients, such as cohort demography, vital signs, and laboratory examinations, were extracted

Data preprocessing mainly included processing missing values, noisy values and duplicate values. The missing value processing process was divided into three aspects: patients, predictors and statistics. We eliminated patients missing more than 30% of their data and predictors missing more than 40%. Because the sampling frequency of each predictor is different and the calculation of statistics such as std, kurt and skew have requirements for sampling frequency, some indicators with very low sampling frequency led to the inability to calculate those statistics. We eliminated the statistics in which the missing data rate was greater than 20% under these indicators. Then, we used mean interpolation to interpolate the remaining missing values. Abnormal values were processed for each predictor. The outliers were found and dealt with by the box-plot combined with the clinical normal range of the different predictors. For example, to protect information about surviving patients older than 90 years old, the age of these patients is recorded as 300 years old. Here, we replaced it with the median value. In addition, duplicate records were deleted, and inconsistent units were converted. For the interval value, we chose the median value to represent the predictor value of the time point. Ultimately, 6,927 admission records remained after preprocessing. Figure 2 shows the patient cohort selection inclusion criteria and the data extraction process, and Table 4 shows the baseline characteristics and outcome measure of our dataset. The median age of the adult patients was 65 years, and 58.8% of patients were male. In-hospital mortality was approximately 19.5%, and the median length of stay in the ICU was 4.7 days. We did not process non-time series predictors such as age and sex. For the time series predictors, we calculated 14 statistics, including min, max, mean, std, median, Q1, Q3, mode, range, IQR, CV, skew, kurt and the first measurement of each predictor from the first 24 hours after admission to the ICU.

**Table 4:** Baseline characteristic and mortality outcome measures. Categorical variables are presented as counts (%), and continuous variables are presented as medians [inter-quartile range Q1-Q3]

	Overall	Died in the hospital	Survived in the hospital
**General**			
Admissions	6927	1350	5577
Age	65.0 [53.0,77.0]	71.0 [58.0,81.0]	64.0 [52.0,75.0]
Gender (Female)	2853 (41.2%)	602 (44.6%)	2251 (40.4%)
First SOFA	6.0 [4.0,8.0]	8.0 [5.0,11.0]	5.0 [4.0,8.0]
First SAPS	21.0 [18.0,25.0]	24.0 [21.0,28.0]	21.0 [18.0,24.0]
First SAPS II	41.0 [32.0,52.0]	53.0 [44.0,64.0]	39.0 [31.0,49.0]
**Origin**			
Elective	1269 (18.3%)	64 (4.7%)	1205 (21.6%)
Emergency	5463 (78.9%)	1237 (91.6%)	4226 (75.8%)
Urgent	195 (2.8%)	49 (3.6%)	146 (2.6%)
**Site**			
MICU	1974 (28.5%)	600 (44.4%)	1374 (24.6%)
SICU	1359 (19.6%)	266 (19.7%)	1093 (19.6%)
CCU	660 (9.5%)	163 (12.1%)	497 (8.9%)
CSRU	1599 (23.1%)	98 (7.3%)	1501 (26.9%)
TSICU	1335 (19.3%)	223 (16.5%)	1112 (19.9%)
**Lab Results**			
HR (BPM)	88.0 [76.0,102.0]	92.0 [78.0,109.0]	87.0 [76.0,100.0]
NBP (MMHG)	113.0 [100.0,130.0]	109.0 [95.0,127.0]	114.0 [101.0,129.0]
RR (CPM)	20.56 [17.71-23.88]	20.78 [17.90-24.19]	20.31 [17.57-23.53]
NA (MMOL/L)	139.0 [137.0,142.0]	140.0 [136.0,143.0]	139.0 [137.0,142.0]
K (MMOL/L)	4.1 [3.8,4.6]	4.2 [3.7,4.7]	4.1 [3.8,4.6]
HCO3 (MMOL/L)	23.0 [19.0,25.0]	21.0 [17.0,24.0]	23.0 [20.0,26.0]
WBC (103/MM3)	11.7 [8.2,16.1]	12.3 [7.5,17.8]	11.7 [8.4,15.8]
P/F Ratio	235.13 [227.10-235.13]	235.13 [207.50-235.23]	235.13 [235.13-235.13]
Ht (%)	28.40 [25.00-32.30]	27.90 [24.40-31.70]	29.10 [25.80-32.70]
Urea (mmol/l)	77.79 [46.35-120.52]	70.27 [34.77-94.43]	104.25 [60.69-140.53]
Bilirubin (mg/dl)	1.00 [0.50-2.36]	1.25 [0.50-2.88]	0.88 [0.45-1.66]
**Outcomes**			
ICU LOS (days)	4.7 [2.4,10.5]	5.7 [2.7,11.7]	4.5 [2.3,10.2]
ICU Death (%)	1129 (16.3%)	1129 (83.6%)	-
Hospital Death (%)	1350 (19.5%)	1350 (100%)	-

### Clinical prediction tasks

The clinical prediction tasks selected in the experiment included patient mortality, length of hospital stay, and disease prediction. Mortality prediction is a primary patient outcome, including short-term, in-hospital and long-term mortality. In the experiment, whether the patient died within 72 hours after entering the ICU was selected as the short-term mortality label, and the 30-day and 1-year mortality rates were used as the long-term mortality label. The length of the hospital stay of an admission can be defined as the time interval between admission and discharge; we calculated the length of hospital stay for each admission in hours. When a patient is discharged, there will be multiple diagnosis, which are represented by the ICD(international statistical classification of disease)-9 diagnosis codes. We followed [10] and divided all the ICD-9 codes into 20 diagnostic groups; each diagnostic group had similar diseases (e.g. respiratory system diagnosis). Thus, the task of disease prediction is transformed into the task of predicting the ICD-9 code groups.

### Experimental design

For the three prediction outcomes, we approximated a global search to obtain the best combination of statistics using a genetic algorithm. To improve the generalizability of the statistical combinations obtained by the genetic algorithm, we embedded the genetic algorithm in a cross-validation procedure, as shown in Table 2. For each data fold, we obtain a set of optimal statistical combinations (i.e., fivefold cross-validation yields 5 sets of statistical combinations). To reduce the effect of randomly partitioning the data during cross-validation, we repeated the entire process five times, selecting different random seeds for dividing the data each time. For the 25 sets of statistical combinations obtained under each prediction task, on the one hand, we compared their prediction performance with the combination of statistic commonly used in previous studies, and on the other hand, we conducted an in-depth analysis of these combinations. Then, we constructed two indexes to quantify the importance of different statistics used for prediction (see the Discussion section). The most important statistics were found by comparing the commonalities and differences of the optimal combination of statistics under different prediction tasks. As performance measures, we choose the AUROC and the area under the precision-recall curve (AUPRC) for the classification tasks and Mean Squared Error (MSE) for the regression tasks. AUROC and AUPRC evaluate the discrimination ability of the model, namely, the ability to assign higher severity scores to patients who died in the hospital compared with those who did not. The higher the AUROC and the AUPRC are, the better the model is. We calculated the mean and standard error of AUROC, AUPRC and MSE scores based on cross-validation as the final result.

All the experiments in this paper were programmed in the Python language, using Spyder 3.6 on a PC equipped with an Intel (R) Core (TM) i7-6700 CPU @ 3.40 GHz processor. The iterations of the genetic algorithm were terminated when the fluctuation in the fitness value became less than *δ*=10^−3^ for 50 consecutive iterations or when the total number of iterations exceeded 200. The crossover probability was set to 0.6, the mutation probability of the genetic algorithm was set to 0.1, and the size of the population was set to 20.

### Results

We report the results under different prediction tasks separately. For each prediction task, we list the prediction results based on a single statistic, commonly used combinations of statistics, and the optimal combinations of statistics obtained by the genetic algorithm.

#### Results of mortality prediction

Patient mortality prediction tasks are divided into short-term, in-hospital, and long-term mortality prediction by survival time. In the experiment, the mortality of patients at 72 hours, in-hospital, 30 days and 1 year were predicted based solely on patients data collected within 24 hours after they entered the ICU. A single statistic can directly reflect the prediction effect. Table 5 shows the AUROC and AUPRC of the 14 selected statistics applied separately for the four mortality prediction tasks. When using a single statistic for mortality prediction, mean, median and Q3 achieved the best results under different prediction tasks. In other words, the statistic that reflects the concentrated trend of the physiological time series achieved the best and near-best prediction results on the mortality prediction task whether in the short or long term prediction. In addition, for short-term mortality prediction, the effect of the max statistic is also significantly greater, which is a statistic that reflects dispersion trends. It is not difficult to understand that if the short-term mortality is predicted using the data of patients 24 hours after entering the ICU, the values that will be significantly related to the predictive label are the degrees of fluctuation of the patient predictors. If the predictors are relatively stable, patient state can also be considered relatively stable. In contrast, large fluctuations are considered to indicate an unstable patient condition; such patients have a higher mortality rate. For the long-term prediction, the average levels of the predictors at a certain stage are closely related to the prediction results over extended periods. If the predictor remain at a consistently abnormal level, the mortality rate is higher over longer time spans.

**Table 5:** Performance of mortality prediction by a single statistic

Statistic	AUROC	AUPRC	AUROC	AUPRC
	72-hour		in-hospital	
min	0.8113 ±0.0023	0.1974 ±0.0040	0.7869 ±0.0028	0.4627 ±0.0073
max	0.8310 ±0.0034	0.2178 ±0.0081	0.8101 ±0.0013	0.4826 ±0.0035
range	0.7789 ±0.0077	0.1782 ±0.0065	0.7622 ±0.0014	0.4188 ±0.0048
mean	**0.8517 ±0.0033**	**0.2282 ±0.0071**	**0.8248 ±0.0016**	**0.5128 ±0.0050**
std	0.7671 ±0.0017	0.1625 ±0.0026	0.7585 ±0.0014	0.4096 ±0.0072
CV	0.7739 ±0.0033	0.1495 ±0.0054	0.7545 ±0.0015	0.4046 ±0.0037
median	0.8498 ±0.0048	0.2191 ±0.0061	0.8234 ±0.0017	0.5091 ±0.0041
Q1	0.8330 ±0.0034	0.2042 ±0.0044	0.8085 ±0.0013	0.4918 ±0.0027
Q3	0.8467 ±0.0010	0.2224 ±0.0089	0.8233 ±0.0020	0.5094 ±0.0036
IQR	0.7463 ±0.0064	0.1287 ±0.0043	0.7447 ±0.0021	0.3854 ±0.0031
mode	0.8395 ±0.0014	0.1995 ±0.0023	0.4804 ±0.0059	
skew	0.6886 ±0.0039	0.0890 ±0.0018	0.7074 ±0.0008	0.3418 ±0.0027
kurt	0.6492 ±0.0118	0.0840 ±0.0042	0.6908 ±0.0018	0.3301 ±0.0025
first	0.7206 ±0.0054	0.1115 ±0.0025	0.7366 ±0.0017	0.3665 ±0.0014
	30-day		1-year	
min	0.7508 ±0.0015	0.5126 ±0.0043	0.7674 ±0.0009	0.7203 ±0.0026
max	0.7671 ±0.0028	0.5237 ±0.0063	0.7790 ±0.0021	0.7289 ±0.0033
range	0.7590 ±0.0018	0.5211 ±0.0059	0.7597 ±0.0013	0.7070 ±0.0028
mean	0.7716 ±0.0033	0.5314 ±0.0049	**0.7838 ±0.0017**	**0.7324 ±0.0034**
std	0.7609 ±0.0027	0.5239 ±0.0053	0.7608 ±0.0033	0.7087 ±0.0049
CV	0.7482 ±0.0025	0.5188 ±0.0033	0.7526 ±0.0011	0.6991 ±0.0022
median	0.7675 ±0.0022	0.5319 ±0.0042	0.7789 ±0.0021	0.7279 ±0.0039
Q1	0.7632 ±0.0027	0.5274 ±0.0047	0.7739 ±0.0017	0.7247 ±0.0023
Q3	**0.7757 ±0.0025**	**0.5368 ±0.0032**	0.7808 ±0.0020	0.7310 ±0.0034
IQR	0.7533 ±0.0037	0.5212 ±0.0024	0.7505 ±0.0016	0.6956 ±0.0016
mode	0.7611 ±0.0022	0.5280 ±0.0066	0.7700 ±0.0022	0.7205 ±0.0023
skew	0.7304 ±0.0020	0.5020 ±0.0033	0.7233 ±0.0023	0.6728 ±0.0054
kurt	0.7210 ±0.0025	0.5017 ±0.0065	0.7196 ±0.0015	0.6781 ±0.0024
first	0.7597 ±0.0036	0.5281 ±0.0069	0.7561 ±0.0018	0.7031 ±0.0028

Table 6 provides the results of mortality prediction by the commonly used combinations of statistics including [mean], [first], [min, max], [min, max, min] and [min, max, mean, std]. Using a single statistic to represent physiological time series obviously leads to information loss and affects the prediction effect. Although the mean performs best as a single statistic, its prediction effect is worse than the prediction effect from combining multiple statistics. The first measurement, which has been used in previous studies, performed the worst; therefore, if only one statistic is used, the first value should not be applied, revealing irrationality in some previous studies. For the different prediction tasks (short-term, in-hospital and long-term mortality), [min, max], [min, max, mean], and [min, max, mean, std] top the list. [min, max, mean] performs best for 72-hour short-term mortality and in-hospital mortality prediction, which shows that the combination of dispersion and central tendency is better. It is further demonstrated that for short-term prediction, statistics that reflect the dispersion tendency have a better representation effect and can reveal fluctuations in the patient’s physiological state. For longer-term mortality prediction tasks (such as 30-day and 1-year), the addition of the std statistic enriches the physiological time series fluctuation information. Even knowing the min, max and mean value of the physiological time series, it is difficult for these statistics to reflect violent fluctuations in the patient’s physiological state. Long-term prediction causes a reduction in the time dependence of the prediction; thus, more information needs to be added to achieve good results.

**Table 6:** Performance of mortality prediction by commonly used combinations of statistics

Statistic	AUROC	AUPRC	AUROC	AUPRC
	72-hour		in-hospital	
mean	0.8517 ±0.0033	0.2282 ±0.0071	0.8248 ±0.0016	0.5128 ±0.0050
first	0.7206 ±0.0054	0.1115 ±0.0025	0.7366 ±0.0017	0.3665 ±0.0014
min, max	0.8590 ±0.0042	0.2558 ±0.0080	0.8308 ±0.0021	0.5289 ±0.0042
min, max, mean	**0.8607 ±0.0021**	**0.2494 ±0.0031**	**0.8310 ±0.0012**	**0.5297 ±0.0030**
min, max, mean, std	0.8589 ±0.0022	0.2498 ±0.0058	0.8282 ±0.0005	0.5262 ±0.0020
	30-day		1-year	
mean	0.7716 ±0.0033	0.5314 ±0.0049	0.7838 ±0.0017	0.7324 ±0.0034
first	0.7597 ±0.0036	0.5281 ±0.0069	0.7561 ±0.0018	0.7031 ±0.0028
min, max	0.7760 ±0.0021	0.5351 ±0.0031	0.7844 ±0.0011	0.7298 ±0.0020
min, max, mean	0.7734 ±0.0017	0.5353 ±0.0041	0.7840 ±0.0022	0.7330 ±0.0049
min, max, mean, std	**0.7770 ±0.0016**	**0.5430 ±0.0058**	**0.7872 ±0.0020**	**0.7391 ±0.0030**

Tables 7, 8, 9, 10 presents the optimal ten combinations of statistics obtained by the genetic algorithm and their performances for short-term, in-hospital and longterm mortality prediction. As shown, the prediction effect of the optimal combination of statistics obtained by the genetic algorithm is rarely weaker than the prediction effect of the commonly used combinations of statistics. As the prediction interval is extended, the prediction performance decreases, which indicates that predicting long-term mortality based only on data collected within 24 hours after patient entering the ICU not ideal. For short-term mortality prediction tasks, Q1 and Q3 appear more frequently. And the statistics that show dispersion tendency also appear frequently, such as min, max and so on. Skew and kurt, two statistics that describe the shape of the time series distribution and are often ignored, appear quite frequently and reflect the role of these two statistics in supplementing the other available information. Under longer-term mortality prediction tasks, mean, Q1 and Q3, which are concentrated statistics, also achieve better results. Combining statistics such as min, max, and mean can better characterize the distribution of physiological time series. In addition, the commonly used combinations of statistics such as [min, max] and [min, max, mean, std] also achieve good prediction results on both in-hospital and long-term mortality prediction tasks. In other words, this paper used experiments to demonstrate why the existing studies chose these particular statistical combinations to represent physiological time series.

**Table 7:** The optimal ten combinations of statistics obtained by the genetic algorithm and their prediction performance for 72-hour mortality prediction

Combination	AUROC	AUPRC
min, max, mean, median, Q1, IQR, kurt	0.8627 ±0.0023	0.2493 ±0.0114
min, max, CV, Q1, Q3, IQR, kurt	0.8620 ±0.0027	0.2398 ±0.0028
min, max, range, mean, Q1, Q3, IQR, skew	0.8609 ±0.0041	0.2427 ±0.0072
min, max, mean, std, Q3	0.8609 ±0.0022	0.2471 ±0.0031
min, std, median, Q1, Q3, skew	0.8607 ±0.0041	0.2444 ±0.0074
min, max, mean	0.8607 ±0.0021	0.2494 ±0.0031
max, mean, Q1, Q3, IQR, kurt	0.8606 ±0.0056	0.2455 ±0.0102
min, mean, std, median, Q1, Q3	0.8605 ±0.0025	0.2485 ±0.0060
min, max, range, mean, CV, Q1, Q3, kurt	0.8604 ±0.0023	0.2454 ±0.0050
min, max, mean, CV, median, Q1, Q3, IQR, kurt	0.8603 ±0.0033	0.2433 ±0.0102

**Table 8:** The optimal ten combinations of statistics obtained by the genetic algorithm and their prediction performances for in-hospital mortality prediction

Combination	AUROC	AUPRC
min, max, range, median	0.8316 ±0.0015	0.5308 ±0.0042
min, max, mean	0.8310 ±0.0012	0.5297 ±0.0030
min, max	0.8308 ±0.0021	0.5289 ±0.0042
min, max, CV, Q1, Q3, kurt, first	0.8285 ±0.0012	0.5268 ±0.0046
max, range, Q1, IQR, mean	0.8280 ±0.0012	0.5236 ±0.0027
range, mean, std, median, Q1, Q3	0.8267 ±0.0008	0.5225 ±0.0025
min, std, Q1, Q3, skew, first	0.8261 ±0.0014	0.5234 ±0.0025
range, mean, std, median, Q1, Q3	0.8257 ±0.0014	0.5216 ±0.0034
min, mean, std, IQR, Q1, kurt	0.8254 ±0.0014	0.5198 ±0.0041
min, range, std, median, Q3, skew, first	0.8253 ±0.0017	0.5225 ±0.0034

**Table 9:** The optimal ten combinations of statistics obtained by the genetic algorithm and their prediction performances for 30-day mortality prediction

Combination	AUROC	AUPRC
min, max, mean, CV, skew, first	0.7780 ±0.0028	0.5351 ±0.0061
min, max, mean, std	0.7770 ±0.0016	0.5430 ±0.0058
mean, std, CV, Q1, min, skew	0.7749 ±0.0027	0.5338 ±0.0058
min, max, std, CV, Q1, Q3, kurt	0.7746 ±0.0015	0.5311 ±0.0061
min, range, std, Q1, Q3, skew, kurt, first	0.7742 ±0.0015	0.5368 ±0.0027
min, range, Q3, skew	0.7741 ±0.0020	0.5348 ±0.0048
max, IQR, kurt	0.7740 ±0.0011	0.5332 ±0.0049
min, range, mean, Q3, IQR	0.7735 ±0.0015	0.5334 ±0.0045
min, max, CV, skew, first	0.7732 ±0.0040	0.5296 ±0.0083
min, max, Q1, Q3, skew	0.7731 ±0.0015	0.5328 ±0.0036

**Table 10:** The optimal ten combinations of statistics obtained by the genetic algorithm and their prediction performance for 1-year mortality prediction

Combination	AUROC	AUPRC
max, mean, std, Q1, kurt	0.7876 ±0.0014	0.7408 ±0.0026
min, max, mean, std	0.7872 ±0.0020	0.7391 ±0.0030
range, mean, std, mode	0.7852 ±0.0020	0.7290 ±0.0021
std, CV, Q1, Q3, skew, kurt	0.7846 ±0.0031	0.7299 ±0.0047
min, max, range, Q1, skew, kurt	0.7846 ±0.0024	0.7347 ±0.0024
range, CV, Q1, Q3, mode, skew, kurt	0.7834 ±0.0012	0.7293 ±0.0031
mean, std, Q1, Q3, skew, first	0.7831 ±0.0010	0.7276 ±0.0013
range, median, Q3, mode, first	0.7823 ±0.0015	0.7263 ±0.0023
range, mean, CV, Q3, skew	0.7821 ±0.0012	0.7275 ±0.0019
max, range, mean, std, CV, median	0.7819 ±0.0013	0.7258 ±0.0016

#### Results of length of hospital stay

Table 11 shows the performance of a single statistic for length of hospital stay prediction. A certain level of correlation exists between the length of hospital stay and mortality prediction. Generally, patients with higher mortality have more severe symptoms; consequently, their hospital stays are relatively long. Consistent with mortality prediction, range works best when based on a single statistic. At the same time, std, CV, and IQR, which reflect the dispersion tendency, have better effects. In addition to indicating the dispersion tendency, the better performing statistics also constitute crossover features, just as *range*=*max*−*min*. Therefore, the importance of cross features is self-evident.

**Table 11:** Performances of single statistics for predicting length of hospital stay

Statistic	MSE
min	59562.72 ±309.59
max	54602.26 ±296.81
range	**47071.82 ±273.31**
mean	58583.26 ±395.47
std	48985.76 ±321.89
CV	50047.97 ±336.91
median	59286.18 ±397.62
Q1	59449.26 ±330.22
Q3	58534.13 ±363.85
IQR	51209.18 ±269.86
mode	60160.49 ±351.09
skew	58055.02 ±333.20
kurt	57259.82 ±178.34
first	61832.78 ±246.62

Table 12 shows the performances of commonly used combinations of statistics for predicting length of hospital stay. [min, max, mean, std] corresponds to the smallest MSE and the best prediction performance. Table 13 shows the optimal ten combinations of statistics obtained by the genetic algorithm and their prediction performances for length of hospital stay prediction. The effect of the combinations of statistics obtained by the genetic algorithm is superior to the effect of the common combinations of statistics. Range appears in each group, illustrating the validity of this statistic for predicting the length of hospital stay of patients. A larger range indicates an unstable condition, and patients with unstable conditions will naturally be hospitalized longer. In contrast, statistics such as the mean, which reflects the central tendency, appear less frequently. When predicting the length of hospital stay, the stability of the patient’s condition is the most important factor; thus, statistics that indicate the dispersion tendencies of time series function better.

**Table 12:** Performances of commonly used combinations of statistics for predicting length of hospital stay

Combination	MSE
mean	58583.26 ±395.47
first	61832.78 ±246.62
min, max	48969.03 ±508.88
min, max, mean	49890.57 ±383.63
min, max, mean, std	**46459.67 ±181.91**

**Table 13:** The optimal ten combinations of statistics obtained by the genetic algorithm and their prediction performances for predicting length of hospital stay

Combination	MSE
min, max, range, std, CV, Q1, Q3, kurt, first	43827.77 ±227.26
min, max, range, CV, median, skew, kurt	43854.10 ±405.73
min, max, range, CV, median, Q3, skew	43854.53 ±297.85
max, range, mean, std, kurt	43868.48 ±314.05
range, CV, Q3, kurt, first	43879.73 ±302.22
min, range, CV, Q3, IQR, skew, kurt, first	43985.47 ±313.01
min, range, CV, Q3, skew, kurt, first	44200.31 ±188.89
min, range, CV, IQR, skew, first	44308.55 ±276.67
max, range, mean, std, Q1, IQR, skew, kurt, first	44318.11 ±323.34
min, max, range, mean, std, median, Q1, Q3, IQR, skew	44334.44 ±301.66

#### Results of disease prediction

We treat disease prediction as a multilabel classification task and calculate the AUROC and AUPRC. Table 14 shows the performances of single statistics for disease prediction. On this task, a comparison of the results shows that the mean, median, Q1, Q3 and other statistics that reflect centralized trends have the best effect. In contrast, the effects of statistics that reflect the dispersion tendency are not very good. The performances of skew and kurt, which reflect the shape of the time series distribution, are the worst. This result shows that if only one statistic is used for patient disease prediction, the shape of the distribution is unimportant; the level of the value is more important.

**Table 14:** Performances of single statistics for disease prediction

statistic	AUROC	AUPRC
min	0.6460 ±0.0097	0.4416 ±0.0131
max	0.6494 ±0.0108	0.4431 ±0.0086
range	0.6371 ±0.0097	0.4208 ±0.0134
mean	**0.6602 ±0.0080**	**0.4470 ±0.0124**
std	0.6271 ±0.0162	0.4230 ±0.0153
CV	0.6173 ±0.0127	0.4094 ±0.0152
median	0.6506 ±0.0073	0.4454 ±0.0110
Q1	0.6503 ±0.0124	0.4396 ±0.0121
Q3	0.6561 ±0.0070	0.4457 ±0.0070
IQR	0.6174 ±0.0142	0.4211 ±0.0106
mode	0.6369 ±0.0128	0.4364 ±0.0138
skew	0.5893 ±0.0159	0.3945 ±0.0110
kurt	0.5915 ±0.0082	0.3923 ±0.0111
first	0.6486 ±0.0158	0.4393 ±0.0082

The corresponding prediction performances of combinations of multiple statistics are shown in Table 15. Among the five commonly used combinations, it is surprising that the single mean statistic works best—even better than combinations of multiple statistics. From the optimal ten combinations obtained by the genetic algorithm shown in Table 16, we can see that the mean statistic appears in almost all the combinations, indicating its core role in disease prediction. Furthermore, min, max, and range are evenly distributed among the multiple combinations. We speculate that these metrics provide good auxiliary data for disease prediction; however, using these statistics alone does not result in good prediction.

**Table 15:** Performances of commonly used combinations of statistics for disease prediction

Combination	AUROC	AUPRC
mean	**0.6602 ±0.0080**	**0.4470 ±0.0124**
first	0.6486 ±0.0158	0.4393 ±0.0082
min, max	0.6558 ±0.0179	0.4488 ±0.0079
min, max, mean	0.6477 ±0.0126	0.4462 ±0.0169
min, max, mean, std	0.6578 ±0.0169	0.4483 ±0.0096

**Table 16:** The optimal ten combinations of statistics obtained by the genetic algorithm and their prediction performances for disease prediction

Combination	AUROC	AUPRC
max, mean, Q3, IQR, first	0.6610 ±0.0088	0.4455 ±0.0127
max, mean, std, Q1, IQR, mode, first	0.6585 ±0.0119	0.4483 ±0.0104
min, range, std, median, Q3, mode, skew, first	0.6581 ±0.0132	0.4462 ±0.0123
range, mean, std, mode	0.6569 ±0.0096	0.4450 ±0.0131
max, std, CV, Q3, IQR, kurt, first	0.6568 ±0.0100	0.4430 ±0.0106
max, range, mean, std, CV, median	0.6565 ±0.0115	0.4442 ±0.0080
range, mean, std, CV, Q1, IQR, skew, first	0.6563 ±0.0068	0.4435 ±0.0100
min, max, mean, std, CV, Q1, IQR, kurt	0.6553 ±0.0141	0.4429 ±0.0105
range, mean, std, median, Q1, Q3	0.6546 ±0.0116	0.4556 ±0.0113
min, mean, std, median, mode	0.6540 ±0.0137	0.4546 ±0.0106

In summary, through the analysis of the prediction performances of different prediction tasks based on single statistics, commonly used combinations of statistics, and approximately optimal combinations of statistics obtained by the genetic algorithm, we discovered many interesting and clinically significant phenomena. We have indirectly demonstrated the rationality of using various combinations of statistics that were applied in previous research. Additionally, we found the statistics that are extremely important in clinical prediction tasks, which can provide guidance for future research.

## Discussion

In the experiments, we used a genetic algorithm to obtain combinations with approximately optimal prediction results for different prediction tasks. Taking 72-hour mortality prediction as an example, the 5-fold cross-validation genetic algorithm was repeated 5 times to obtain 25 groups of combinations. Each group corresponds to multiple statistics, and the prediction performance varies among the different combinations. Which statistics appear most frequently and which statistics will achieve better prediction results are meaningful research questions. In the previous chapter, we performed a rough analysis. In this chapter, we quantitatively analyse the frequency of each statistic in the optimal combinations and the mean values of indexes under different tasks. Since we chose random forest as the classifier in the experiments, it is necessary to verify the performances of other classifiers based on the obtained statistics. So we also discuss this issue.

Tables 17, 18, and 19 show the results of each statistic regarding patient mortality, length of hospital stay and disease prediction, respectively. Frequency represents the number of occurrences of a statistic in the 25 combinations, and Mean_AUROC and Mean_AUPRC represent the average AUROC and AUPRC for all the combinations in which the statistic appears.

**Table 17:** Quantitative analysis results of each statistic for mortality prediction

Statistic	Frequency	Mean_AUROC	Mean_AUPRC	Frequency	Mean_AUROC	Mean_AUPRC
	72-hour			In-hosp		
min	**22**	0.8598	0.2447	**14**	**0.8262**	**0.5221**
max	**22**	0.8597	0.2446	12	**0.8264**	**0.5222**
range	7	0.8592	0.2429	8	0.8258	0.5215
mean	17	0.8598	0.2453	12	0.8253	0.5197
std	10	0.8594	0.2459	**14**	0.8245	0.5195
CV	9	0.8596	0.2433	8	0.8242	0.5187
median	5	**0.8605**	0.2461	13	0.8249	0.5205
Q1	20	**0.8599**	0.2440	11	0.8255	0.5208
Q3	20	0.8597	0.2438	13	0.8249	0.5201
IQR	12	0.8599	0.2443	9	0.8245	0.5191
mode	3	0.8592	0.2463	7	0.8235	0.5179
skew	5	0.8596	0.2429	13	0.8241	0.5192
kurt	14	0.8598	0.2443	12	0.8244	0.5180
first	3	0.8584	0.2429	11	0.8248	0.5192
	30-day			1-year		
min	**17**	**0.7726**	**0.5323**	5	**0.7830**	**0.7311**
max	14	0.7721	0.5305	13	0.7819	0.7280
range	12	0.7710	0.5306	12	0.7817	0.7269
mean	14	0.7718	0.5312	**16**	0.7819	0.7274
std	12	**0.7722**	**0.5314**	**17**	0.7820	0.7272
CV	12	0.7719	0.5305	12	0.7813	0.7258
median	10	0.7704	0.5289	7	0.7810	0.7259
Q1	14	0.7712	0.5302	15	0.7819	0.7275
Q3	12	0.7715	0.5305	10	0.7816	0.7262
IQR	11	0.7708	0.5298	8	0.7802	0.7242
mode	12	0.7699	0.5285	10	0.7814	0.7262
skew	**17**	0.7717	0.5308	13	0.7815	0.7264
kurt	10	0.7718	0.5301	9	**0.7827**	**0.7291**
first	9	0.7718	0.5309	10	0.7809	0.7253

**Table 18:** Quantitative analysis results of each statistic for predicting length of hospital stay

Statistic	Frequency	Mean_MSE
min	15	44758.80
max	14	44706.94
range	**18**	**44440.71**
mean	12	44979.37
std	11	44800.35
CV	14	44770.15
median	10	44894.44
Q1	11	45090.02
Q3	15	44772.27
IQR	11	44770.07
mode	7	45325.18
skew	16	44670.12
kurt	14	44678.53
first	13	44771.67

**Table 19:** Quantitative analysis results of each statistic for disease prediction

Statistic	Frequency	Mean_AUROC	Mean_AUPRC
min	6	0.6541	0.4447
max	14	0.6544	0.4443
range	13	0.6542	0.4464
mean	13	**0.6550**	0.4464
std	**16**	0.6547	0.4454
CV	9	0.6541	0.4434
median	11	0.6538	**0.4476**
Q1	13	0.6539	0.4459
Q3	11	0.6543	**0.4467**
IQR	10	**0.6550**	0.4436
mode	9	0.6539	0.4450
skew	8	0.6534	0.4435
kurt	8	0.6532	0.4441
first	11	0.6541	0.4451

In the mortality prediction task, the statistics with the highest frequency for 72-hour short-term mortality prediction are min, max, Q1 and Q3. The mean_AUROC and mean_AUPRC values corresponding to median and Q1 are high, while first are low. Statistics that embody the dispersion tendency, such as min and max, play a central role in short-term mortality prediction, while statistics such as first are more irrelevant to patients’ physiological status information. For the in-hospital mortality prediction task, min and std occurred most frequently, and min and max achieved the highest Mean_AUROC and Mean_AUPRC, respectively. For the long-term mortality prediction task, min, std, and kurt performed best. Kurtosis and skew measures have rarely been used in previous studies to measure the shapes of physiological time series distributions. However, the experiments in this paper show that these two statistics provide supplementary information and should not be discarded. Apart from this lack, we can clearly see that the statistics widely used in previous studies have indeed played a better role in predicting mortality. When predicting the length of hospital stay, range appears most often, and its effect is the best. In the disease prediction task, the most frequent occurrence is std, but the measures that perform the best are statistics that reflect the central tendency.

To verify whether the combinations of statistics obtained in this paper can also obtain good prediction results using other classifiers, we select logistic regression, SVM and decision tree. We compare the prediction performance of the optimal combination of statistics and the commonly used combinations of statistics under different prediction tasks by multiple classifiers. Tables 20, 21, 22 and 23 show the results of the 72-hour, in-hospital, 30-day, and 1-year mortality prediction, respectively. Tables 24 and 25 show the results of the length of hospital stay and the disease group prediction.

**Table 20:** Performance of 72-hour mortality prediction by multiple classifiers

Combination	AUROC	AUPRC	AUROC	AUPRC
	Logistic regression		Random forest	
mean	0.8356 ±0.0014	0.2277 ±0.0037	0.8517 ±0.0033	0.2282 ±0.0071
first	0.7147 ±0.0030	0.1440 ±0.0031	0.7206 ±0.0054	0.1115 ±0.0025
min, max	0.8374 ±0.0019	0.2395 ±0.0031	0.8590 ±0.0042	0.2558 ±0.0080
min, max, mean	0.8475 ±0.0023	0.2469 ±0.0044	0.8607 ±0.0021	0.2494 ±0.0031
min, max, mean, std	**0.8484 ±0.0022**	**0.2506 ±0.0065**	0.8589 ±0.0022	0.2498 ±0.0058
min, max, mean, median, Q1, IQR, kurt	0.8426 ±0.0029	0.2377 ±0.0039	**0.8627 ±0.0023**	**0.2493 ±0.0114**
	SVM		Decision tree	
mean	0.8307 ±0.0031	0.2191 ±0.0080	**0.6050 ±0.0164**	**0.0778 ±0.0066**
first	0.6950 ±0.0082	0.1147 ±0.0074	0.5356 ±0.0163	0.0572 ±0.0050
min, max	0.8345 ±0.0031	0.2163 ±0.0041	0.5972 ±0.0112	0.0795 ±0.0052
min, max, mean	0.8372 ±0.0042	0.2158 ±0.0035	0.6040 ±0.0165	0.0835 ±0.0082
min, max, mean, std	0.8331 ±0.0043	0.2196 ±0.0088	0.5895 ±0.0118	0.0752 ±0.0054
min, max, mean, median, Q1, IQR, kurt	**0.8377 ±0.0023**	**0.2275 ±0.0058**	0.5952 ±0.0121	0.0777 ±0.0058

**Table 21:** Performance of in-hospital mortality prediction by multiple classifiers

Combination	AUROC	AUPRC	AUROC	AUPRC
	Logistic regression		Random forest	
mean	0.8122 ±0.0040	0.5147 ±0.0021	0.8248 ±0.0016	0.5128 ±0.0050
first	0.7354 ±0.0033	0.4019 ±0.0022	0.7366 ±0.0017	0.3665 ±0.0014
min, max	0.8277 ±0.0041	0.5328 ±0.0020	0.8308 ±0.0021	0.5289 ±0.0042
min, max, mean	0.8301 ±0.0034	0.5365 ±0.0016	0.8310 ±0.0012	0.5297 ±0.0030
min, max, mean, std	0.8315 ±0.0018	0.5416 ±0.0010	0.8282 ±0.0005	0.5262 ±0.0020
min, max, range, median	**0.8330 ±0.0022**	**0.5429 ±0.0013**	**0.8316 ±0.0015**	**0.5308 ±0.0042**
	SVM		Decision tree	
mean	0.7997 ±0.0018	0.5121 ±0.0037	0.6163 ±0.0014	0.2652 ±0.0012
first	0.7190 ±0.0029	0.3779 ±0.0023	0.5797 ±0.0039	0.2359 ±0.0031
min, max	0.8056 ±0.0026	0.5237 ±0.0018	0.6243 ±0.0039	0.2724 ±0.0024
min, max, mean	0.8124 ±0.0026	0.5377 ±0.0026	0.6256 ±0.0015	0.2739 ±0.0024
min, max, mean, std	0.8165 ±0.0019	0.5424 ±0.0017	0.6241 ±0.0041	0.2719 ±0.0027
min, max, range, median	**0.8186 ±0.0026**	**0.5446 ±0.0015**	**0.6337 ±0.0030**	**0.2800 ±0.0024**

**Table 22:** Performance of 30-day mortality prediction by multiple classifiers

Combination	AUROC	AUPRC	AUROC	AUPRC
	Logistic regression		Random forest	
mean	0.7257 ±0.0024	0.5118 ±0.0005	0.7716 ±0.0033	0.5314 ±0.0049
first	0.7321 ±0.0029	0.5139 ±0.0027	0.7597 ±0.0036	0.5281 ±0.0069
min, max	0.7376 ±0.0022	0.5140 ±0.0018	0.7760 ±0.0021	0.5351 ±0.0031
min, max, mean	0.7380 ±0.0035	0.5148 ±0.0038	0.7734 ±0.0017	0.5353 ±0.0041
min, max, mean, std	**0.7404 ±0.0017**	**0.5184 ±0.0034**	0.7770 ±0.0016	0.5430 ±0.0058
min, max, mean, CV, skew, first	0.7403 ±0.0041	0.5178 ±0.0045	**0.7780 ±0.0028**	**0.5351 ±0.0061**
	SVM		Decision tree	
mean	0.7322 ±0.0015	0.5162 ±0.0013	0.5778 ±0.0029	0.3550 ±0.0010
first	0.7164 ±0.0012	0.5055 ±0.0046	0.5102 ±0.0035	0.3069 ±0.0018
min, max	0.7282 ±0.0011	0.5034 ±0.0041	0.5566 ±0.0010	0.3444 ±0.0048
min, max, mean	0.7395 ±0.0033	0.5121 ±0.0046	0.5756 ±0.0034	0.3536 ±0.0012
min, max, mean, std	0.7218 ±0.0024	0.5039 ±0.0013	**0.5867 ±0.0003**	**0.3619 ±0.0005**
min, max, mean, CV, skew, first	**0.7454 ±0.0015**	**0.5271 ±0.0035**	0.5831 ±0.0026	0.3592 ±0.0024

**Table 23:** Performance of 1-year mortality prediction by multiple classifiers

Combination	AUROC	AUPRC	AUROC	AUPRC
	Logistic regression		Random forest	
mean	0.7704 ±0.0025	0.7398 ±0.0030	0.7838 ±0.0017	0.7324 ±0.0034
first	0.7505 ±0.0043	0.7171 ±0.0024	0.7561 ±0.0018	0.7031 ±0.0028
min, max	0.7759 ±0.0036	0.7404 ±0.0046	0.7844 ±0.0011	0.7298 ±0.0020
min, max, mean	0.7776 ±0.0033	0.7413 ±0.0015	0.7840 ±0.0022	0.7330 ±0.0049
min, max, mean, std	0.7899 ±0.0024	0.7534 ±0.0040	0.7872 ±0.0020	0.7391 ±0.0030
max, mean, std, Q1, kurt	**0.7998 ±0.0007**	**0.7642 ±0.0028**	**0.7876 ±0.0014**	**0.7408 ±0.0026**
	SVM		Decision tree	
mean	0.7996 ±0.0015	0.7634 ±0.0018	0.6367 ±0.0019	0.5653 ±0.0037
first	0.7694 ±0.0024	0.7343 ±0.0026	0.6218 ±0.0006	0.5495 ±0.0024
min, max	0.7923 ±0.0024	0.7552 ±0.0024	0.6368 ±0.0026	0.5654 ±0.0013
min, max, mean	0.7959 ±0.0023	0.7607 ±0.0021	0.6375 ±0.0030	0.5658 ±0.0014
min, max, mean, std	0.7978 ±0.0019	0.7619 ±0.0017	**0.6409 ±0.0028**	**0.5689 ±0.0023**
max, mean, std, Q1, kurt	**0.8090 ±0.0013**	**0.7734 ±0.0007**	0.6370 ±0.0018	0.5654 ±0.0028

**Table 24:** Performance of length of hospital stay prediction by multiple classifiers

Combination	Logistic regression	Random forest
mean	87295.21 ±136.38	58583.26 ±395.47
first	85075.99 ±141.76	61832.78 ±246.62
min, max	83285.19 ±248.05	48969.03 ±508.88
min, max, mean	81878.83 ±348.28	49890.57 ±383.63
min, max, mean, std	80245.68 ±234.80	46459.67 ±181.91
min, max, range, std, CV, Q1, Q3, kurt, first	**75391.36 ±642.39**	**43827.77 ±227.26**

**Table 25:** Performance of disease prediction by multiple classifiers

Combination	AUROC	AUPRC	AUROC	AUPRC
	Logistic regression		Random forest	
mean	**0.6537 ±0.0073**	**0.5251 ±0.0053**	0.6602 ±0.0080	0.4470 ±0.0124
first	0.6229 ±0.0055	0.4932 ±0.0069	0.6486 ±0.0158	0.4393 ±0.0082
min, max	0.6395 ±0.0054	0.5053 ±0.0055	0.6558 ±0.0179	0.4488 ±0.0079
min, max, mean	0.6509 ±0.0123	0.5203 ±0.0115	0.6477 ±0.0126	0.4462 ±0.0169
min, max, mean, std	0.6483 ±0.0084	0.5153 ±0.0077	0.6578 ±0.0169	0.4483 ±0.0096
max, mean, Q3, IQR, first	0.6521 ±0.0081	0.5262 ±0.0077	**0.6610 ±0.0088**	**0.4455 ±0.0127**
	SVM		Decision tree	
mean	0.6407 ±0.0109	0.4399 ±0.0120	**0.5267 ±0.0032**	**0.3329 ±0.0030**
first	0.6370 ±0.0083	0.4281 ±0.0087	0.5203 ±0.0079	0.3291 ±0.0053
min, max	0.6399 ±0.0067	0.4293 ±0.0069	0.4983 ±0.0109	0.3020 ±0.0021
min, max, mean	0.6407 ±0.0109	0.4407 ±0.0099	0.5196 ±0.0110	0.3203 ±0.0063
min, max, mean, std	**0.6437 ±0.0054**	**0.4426 ±0.0064**	0.5234 ±0.0070	0.3281 ±0.0083
max, mean, Q3, IQR, first	0.6401 ±0.0115	0.4374 ±0.0073	0.5201 ±0.0119	0.3266 ±0.0086

In the task of mortality prediction, regardless of short-term, in-hospital or long-term prediction, from a horizontal perspective, the decision tree has a poor prediction effect. The performance of SVM is similar to random forest, but the time complexity is high. Logistic regression is usually able to achieve higher AUPRC. The time complexity of the random forest is low, and it can obtain the best prediction effect in most cases compared to other classifiers. This is why we choose the random forest as the classifier at the stage of calculating the fitness value by the genetic algorithm. Vertically, patient representation based on the best combination of statistics has achieved the best prediction results in most cases compared to the commonly used combinations of statistics. A single statistic such as mean and first is less effective than the combination of multiple statistics. In the cases where the optimal combination of statistics does not achieve the optimal effect, the combination of [min, max, mean, std] has achieved the optimal effect many times. On the one hand, it shows that the statistical combinations obtained by random forest and the analysis of effective statistics are also applicable to other classifiers. On the other hand, it also reflects the scientific nature of the commonly used combinations of statistics such as [min, max, mean, std].

In the length of stay prediction task, the MSE of random forest is much smaller than the MSE of logistic regression. The MSE corresponding to the optimal combination is smaller than the commonly used combination, and much smaller than the MSE corresponding to a single statistic. In the disease prediction task, the optimal combination of statistics only performs best when the random forest is used as a classifier. When logistic regression and decision tree are used as classifiers, the performance based on a single statistic ’mean’ is the best. Although the optimal combination of statistics do not achieve the best prediction effect, in the results of random forest, we can also find that the effect of mean and optimal combination of statistics is not much different. It is also consistent with the conclusion that the statistic ’mean’ plays an important role in disease prediction. In general, the effective statistical combinations based on random forest in this paper can also achieve better prediction results when selecting other classifiers. It shows that the discussion of effective statistics under different prediction tasks in this paper has a strong generalization ability.

## Conclusion

In this paper, we summarized 14 statistics that describe the characteristics of physiological time series, of which three involve aspects of the central tendency, dispersion tendency, and distribution shape. Then, we evaluated the performances of these summary statistics of physiological time series as features for clinical prediction tasks, including patient mortality, length of hospital stay and disease prediction. We performed experiment on patient representations based on both single statistics and commonly used combinations of statistics. To find the combinations of statistics with the best prediction performances under different tasks (limited by the high time complexity of global search), we used a cross-validation-integrated with a genetic algorithm to obtain the combinations of statistics with approximately optimal performances. A quantitative analysis was performed on each statistic in the optimal combinations. Through in-depth analysis of the experimental results, we have reached the following conclusions: (1) As the prediction time becomes longer, the prediction performance becomes increasingly worse. Using data acquired only within 24 hours after the patient entered the ICU was insufficient to make reasonable long-term mortality prediction. (2) Statistics that reflect centralized trends, such as mean and median, play an important role in almost all mortality prediction tasks. (3) For short-term mortality prediction, statistics that show dispersion tendency are also representative, such as min, and max. Cross-features such as range may contain more information. (4) For the length of hospital stay prediction task, the statistics that reflect the dispersion tendency perform better. The length of hospital stay is closely related to the stability of the patient’s physiological state: unstable patients have a higher probability of staying longer. (5) For the disease prediction task, statistics that reflect the centralized trend, such as the mean, make larger contributions to the prediction result.

The mean represents the average level of different predictors is sig- nificantly correlated with judgements concerning whether the patient’s condition is due to a specific disease. (6) Commonly used combinations of statistics such as [min, max, mean] and [min, max, mean, std] achieve good prediction results in most cases; thus, these experiments help to verify the rationality of previous research. (7) Skew and kurt, which reflect the shape of a distribution, perform poorly when used individually as features for prediction, but they appear frequently in the optimal combinations, indicating that they can play a role as supplemental information.

Although we evaluated the effect of statistics of physiological time series under different prediction tasks, some limitations still exist. This paper considers the central tendency, dispersion tendency and distribution shape when choosing statistical features but does not fully consider latent characteristics, such as periodicity. Moreover, due to limitations in the sampling frequencies of some of the clinical predictors, the analysis of kurt and skew, which describe shape of a distribution, was insufficient. Furthermore, these experiments were applied only to patient mortality, length of hospital stay and disease prediction. Research on other clinical tasks still needs to be performed. In future work, we plan to correct the deficiencies of this study and design a more suitable patient representation method and model to improve the results of clinical task prediction.

## Data Availability

The data (MIMIC-III Dataset) used in our experiment can be obtained from https://mimic.physionet.org/. Researchers seeking to use the database must formally request access following the steps listed on that website.

## Abbreviations

APACHE: Acute physiology and chronic health evaluation
AUPRC: Area under the precision-recall curve
AUROC: Area under the receiver operating characteristic curve
Bun: Urea nitrogen
CV: Coefficient of variation
EHR: Electronic health record
GCS: Glasgow coma scale
HR: Heart rate
ICD: International statistical classification of disease
ICU: Intensive care unit
IQR: Interquartile range
kurt: kurtosis
max: Maximum
MIMIC: Medical information mart for intensive care
min: Minimum
MODS: Multiple organ dysfunction syndrome
SAPSII: New simplified acute physiology score
SBP: Systolic blood pressure
Q1: Lower quartile
Q3: Upper quartile
skew: Skewness
SOFA: Sepsis-related organ failure assessment
SOI: Severity of illness
std: Standard variation
